# Seroprevalence of pertussis in Madagascar and implications for vaccination

**DOI:** 10.1017/S0950268820002800

**Published:** 2020-11-16

**Authors:** Solohery L. Razafimahatratra, Amy Wesolowski, Lala Rafetrarivony, Jean-Michel Heraud, Forrest K. Jones, Simon Cauchemez, Richter Razafindratsimandresy, Sandratana J. Raharinantoanina, Aina Harimanana, Jean Marc Collard, C. J. E. Metcalf

**Affiliations:** 1 Bacteriology Experimental Unit, Institut Pasteur de Madagascar, Antananarivo, Madagascar; 2 Department of Epidemiology, Johns Hopkins Bloomberg School of Public Health, Baltimore, MD, USA; 3 Virology Unit, Institut Pasteur de Madagascar, Antananarivo, Madagascar; 4 Mathematical Modelling of Infectious Diseases Unit, Institute Pasteur, UMR 2000, CNRS, Paris, France; 5 Epidemiology and Clinical Research Unit, Institut Pasteur de Madagascar, Madagascar; 6 Department of Ecology and Evolutionary Biology and Princeton School of Public and International Affairs, Princeton University, Princeton, NJ, USA

**Keywords:** Madagascar, pertussis, serology, vaccination

## Abstract

Pertussis is a highly contagious infectious disease and remains an important cause of mortality and morbidity worldwide. Over the last decade, vaccination has greatly reduced the burden of pertussis. Yet, uncertainty in individual vaccination coverage and ineffective case surveillance systems make it difficult to estimate burden and the related quantity of population-level susceptibility, which determines population risk. These issues are more pronounced in low-income settings where coverage is often overestimated, and case numbers are under-reported. Serological data provide a direct characterisation of the landscape of susceptibility to infection; and can be combined with vaccination coverage and basic theory to estimate rates of exposure to natural infection. Here, we analysed cross-sectional data on seropositivity against pertussis to identify spatial and age patterns of susceptibility in children in Madagascar. A large proportion of individuals surveyed were seronegative; however, there were patterns suggestive of natural infection in all the regions analysed. Improvements in vaccination coverage are needed to help prevent additional burden of pertussis in the country.

## Introduction

Pertussis is a highly contagious infectious disease caused by the bacterium *Bordetella pertussis*. Although there is an effective and widely implemented infant vaccination programme globally, pertussis still causes an estimated 24.1 million cases and ~160 000 deaths in children less than 5 [1–3]. Outbreaks of the disease remain common, with resurgences even in high-income countries with high vaccination coverage [4, 5]. In 2014, vaccination coverage with the three doses of pertussis-containing vaccine required for effective immunisation was estimated at 86% globally [6]; but there is considerable heterogeneity both nationally and sub-nationally [2]. However, national and sub-national heterogeneity in coverage and the subsequent outbreak risk are poorly characterised, particularly in low-income settings.

In many resource-limited settings, accurate individual-level vaccination coverage data are unavailable; and mortality and morbidity estimates are rare and uncertain, as individuals infected with pertussis may not seek care, particularly since symptoms are less severe in vaccinated individuals [7–9] and diagnostic methods to detect *B. pertussis* are often in limited use [10]. This lack of data means that burden estimates are often based on models that rely on vaccine coverage estimates, themselves subject to uncertainty [11]. The result is important gaps in our understanding of the burden of pertussis, effectiveness of vaccination programmes and pertussis outbreak risk.

Since pertussis is a difficult disease to monitor using traditional surveillance methods, serological surveillance might provide a useful alternative approach to probing the dynamics and burden of the infection. Around 80–85% of individuals develop antibodies to the pertussis toxin (PT) at concentrations >100 UI/ml following pertussis infection; around 78% do so following vaccine with the whole-cell pertussis vaccine (our focus here), although this shows considerable variability [6]. Serological titres peak briefly after natural infection (with titres exceeding 100 UI/mL); and has been observed to wane following both vaccination and natural infection, which may impede progress towards achieving high population-level immunity to reduce the incidence of infections [12]. Although serological titres do not map perfectly to immuno-protection [6, 12], they can still be used to obtain an indication of the landscape of susceptibility. Measures of seropositivity combined with mathematical models can also be used to estimate the baseline force of infection to provide an indication of the dynamics of natural infection [13–17].

Madagascar introduced pertussis vaccination in 1976 as part of the Expanded Program on Immunisation (EPI), and nationally reported cases have fallen substantially since then [13, 18]. Vaccination became routine in the late 1980s and widely available as part of routine activities in all health facilities across the country. Currently, Madagascar administers three doses of the DTCHepB + Hib vaccination (whole-cell vaccine) which follows the WHO recommended vaccination schedule at 6, 10 and 14 weeks [19]. Coverage for all three doses at the country level has been estimated to vary between 83% and 93% during the 2008–2017 period [19, 20], although heterogeneities in coverage exist at the district level. Limited reporting further complicates our understanding of pertussis epidemiology. Pertussis is not included in the list of notifiable diseases in Madagascar and few cases are laboratory confirmed since there is only a single laboratory (Institut Pasteur de Madagascar) in the country which has recently acquired these capabilities. Given the uncertainties in pertussis epidemiology in Madagascar, we analysed serological profiles of anti-PT IgG in a cross-section of Malagasy children from five different sites in order to assess the current immunization programme and circulation of natural infection.

## Materials and methods

### Sera collection

**Fig. 1.**
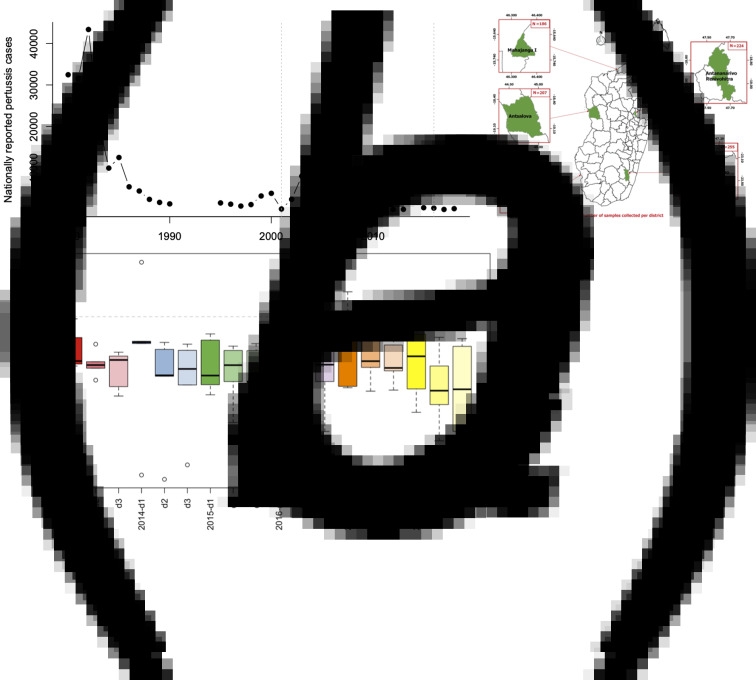
Epidemiology of pertussis and DTP vaccination in Madagascar. (a) Nationally reported pertussis cases from the WHO indicate consistently low case numbers after 2004; vertical dashed lines indicate the time-range experienced by individuals within our cross-sectional seroepidemiological sample that occurred in 2016 and reached children aged between 9 months and 15 years (2001–2016). (b) Location of the five districts where the study took place (shown in green) across Madagascar, with sample sizes of each shown in the insets. (c) Vaccination coverage (number of doses given over the estimated target population) for DTP 1–3 nationally and for the five districts studied. Mean coverage decreased by the dose number and was highly variable amongst the districts (DTP1 = 83%, min = 30%, max = 124%; DTP2 = 75%, min = 28%, max = 98%; DTP3 = 74%, min = 34%, max = 95%).

Serum samples were collected from May to September in 2016 as part of a separate study to estimate the seroprevalence of poliovirus antibodies in high-risk areas for vaccine-derived poliovirus (VDPV) outbreaks in Madagascar [20]. Written consent was provided by participants for the use of their serum to analyse other infectious diseases. Five sites were originally chosen based on reported VDPV outbreaks and consistently high performance in the acute flaccid paralysis case surveillance (see Fig. 1b). The sites included were Mahajanga (CUM: Urban commune of Mahajanga), Toliary I, Antsalova, Midongy Atsimo and Antananarivo (CUA: Urban commune of Antananarivo). These districts represent multiple regions of the country and a rural/urban and population gradient. The districts represent a range of population densities from more populated areas Antananarivo (CUA in the Analamanga Region) at 213.95 individuals per square kilometre down to the sparsely populated Antsalova in the west coast of the country with only 7.97 individuals per square kilometre (Midongy Atsimo, Atsimo-Atsinanana region in the southeast: 54.43 individuals per square kilometre, Toliary I, Atsimo-Andrefana region in the southwest: 27.16 individuals per square kilometre, and CUM, Boeny region in the northwest with 29.99 individuals per square kilometre). These districts also cover regions in the country with large proportions of their population in the lowest wealth brackets in the country.

For each participant, 2 ml of blood were collected, allowed to clot before being separated by centrifugation and transported to the Institut Pasteur de Madagascar (IPM) in Antananarivo. Individuals aged 6 months to 15 years were eligible for inclusion. Samples were stored at −80 °C at IPM. In total, we tested 1078 samples across the five districts (255 samples from Midongy Atsimo, 224 samples from CUA, 207 from Antsalova, 206 samples from Toliary I and 186 from CUM (see Table 1)). The following age groups were separately analysed: 6–11 months, 3–4, 5–6, 7–8, 9, 10, 11, 12–13 and 14–15 years (see Table 1, Fig. 1a).

**Table 1.** Distribution of sera collected in five districts of Madagascar by age groups

Age groups	Districts					Total
	Antsalova	CUA	CUM	Midongy Atsimo	Toliary I	
6–11m	51	58	14	85	20	228
3–4y	66	76	74	88	90	394
5–6y	16	12	10	11	15	64
7–8y	20	12	20	13	11	76
9y	8	8	7	12	6	41
10y	9	14	10	10	18	61
11y	11	10	8	7	8	44
12–13y	16	17	24	19	14	90
14–15y	10	17	19	10	24	80
Total	207	224	186	255	206	1078

CUA, Urban Commune of Antananarivo; CUM, Urban Commune of Mahajanga; m, months; y, years.

### Laboratory methods

The level of anti-PT IgG (IU/ml) is a marker of either pertussis infection or vaccination and is specific for *B. pertussis* [20–22]. The presence of anti-PT IgG was detected by quantitative analysis using a commercially available ELISA kit (EUROIMMUN, Lübeck, Germany). The EUROIMMUN kit has a high specificity and sensitivity compared to the other commercialised kits summarised in the Annex Table. Although this kit is commonly used for diagnosis, it has previously been used to estimate seroprevalence [23]. All experiments were conducted at the laboratories of Experimental Bacteriology of the Institut Pasteur de Madagascar (IPM) according to the manufacturer's manual. The lower and upper limits of detection for the kit were 5 and 200 IU/ml of anti-PT IgG, respectively. Anti-PT IgG levels exceeding 200 IU/ml were reported as 201 IU/ml; no re-examination was performed to obtain the exact values. Level 100 IU/ml indicated acute pertussis infection or recent vaccination, while 40–100 IU/ml were interpreted as recent exposure to pertussis. Level of <40 IU/ml was interpreted as no evidence of acute infection [24, 25]. The proportions with anti-PT IgG levels of <5, 5–40, 40–100 and 100 IU/ml were also assessed.

### Statistical methods

Data were analysed using R v3.6.0. Plots of titres across age and space were used to evaluate the distribution of data, and non-parametric statistical tests were used to identify regions or age groups that differed significantly (e.g. the two-sided Mann–Whitney for continuous variables, Fisher's exact test for categorical variables). Aggregated data are available upon request and at https://github.com/apwez/Pertussis_Madagascar.

### Estimating the force of infection across years

The data consist of age and IgG serostatus for individuals sampled in 2016 across ages and locations, including seropositive individuals (defined as *Y_i,t,j_* = 1, corresponding to a quantitative titre >5 IU/ml, where *i* indexes individual observations, *t* indexes the year, and *j* the location) and seronegative individuals (*Y_i,t,j_* = 0). To be seropositive at any age requires having been infected at some prior age, or vaccinated. In the absence of vaccination, if the force of infection, *l_j,t_*, captures the rate at which susceptible individuals become infected in location *j* in year *t*, we can use a catalytic model to frame the probability that an individual *i* is immune at age *a* in year *y* [26] as:
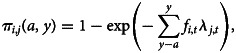
where *a* is the age of the focal individual in year of measurement *y*, and *f_i,t_* reflects the fraction of exposure in year *t* that individual experienced, which will be <1 for individuals sampled part way through that year, or individuals still protected by maternal immunity (assumed negligible here, since all individuals are aged >6 months). This framing assumes that seropositivity maps to immunity; and that waning of seropositivity (and immunity) is negligible over our focal time frames, neither of which may perfectly reflect the biology of pertussis. However, seropositivity is the best measure of immunity currently available, so the former is a necessary first approximation; while the latter is likely to be adequate over the time-scales considered here, as the duration of seropositivity (following both natural infection and three doses with the whole-cell vaccine, see below) has been shown to be at least 9 years [27]. The framing above also neglects the fact that the force of infection might also vary over age (as a result of, e.g. different pattern of contact over age). Variation over both age and time are difficult to identify with a single cross-sectional survey, and variation over age is likely of a much smaller magnitude than variation in the force of infection through time given increases in vaccination coverage and declines in pertussis cases over the life-span of individuals in the study (Fig. 1a). We therefore assume that the force of infection does not vary systematically by age.

Since all individuals aged >6 months should have had the opportunity of experiencing the full course of pertussis vaccination (i.e. all three doses), we need to extend the expression of the probability of being seronegative to encompass both having missed the opportunity of becoming seropositive via vaccination, and having subsequently evaded infection. The probability of being seropositive becomes:
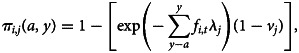


that is, in each location, we also incorporate the probability of becoming seropositive as a result of vaccination. Vaccination does not necessarily translate into seropositivity (given varying efficacy, etc.), so our estimates of *v_j_* should be lower than levels of vaccination coverage [28]. We also assume a constant probability of vaccination in each location (rather than, e.g. improvements through time), and that in children aged <6 months, vaccination is the only source of seropositivity, which could include delivery both via the routine programme and via vaccination weeks. This assumption has the potential to underestimate the risk of infection in the youngest age classes. Emerging potential biases are further discussed below.

The serostatus of individuals at a given time-point reflects their exposure in all the years up to that time-point. For each location, we maximise the binomial likelihood of observations *Y_i,j_* across all individuals given the probability of being immune *π*_*i*,*j*_(*a*, *y*):
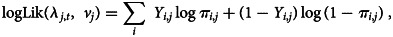
obtaining estimates of the force of infection *λ_j_* for each location *j* and year *t* and the probability of becoming seropositive as a result of vaccination *v*_*j*_ for each location *j*. We fit the model using a Markov Chain Monte Carlo approach with the programme RStan, assuming weak priors on the probability of vaccination, *v_j_*, running four parallel chains and checking for convergence.

## Results

Few pertussis cases were reported annually in recent years according to the WHO (Fig. 1a) [29]. This may reflect successful immunization by vaccination or limited availability of molecular diagnostics (requires a PCR test) and surveillance. Administrative estimates of national vaccination coverage for the three doses of the pertussis vaccine (delivered in a schedule combined with diphtheria and tetanus) were calculated by dividing doses distributed by the estimated target population. For the districts studied, coverage for the first dose was on average 83% (min = 30%, max = 124%), for the second dose was 75% (min = 28%, max = 98%) and for the third dose was 74% (min = 34%, max = 95%) (Fig. 1c). However, there is substantial geographic heterogeneity in this measure; as well as uncertainty in how many individuals have completed their complete dosing schedule (which may be much lower than the aggregate numbers of doses delivered, as the same individuals may be vaccinated repeatedly). As a result, it is hard to evaluate the distribution of individuals protected by vaccination from these data.

**Fig. 2.**
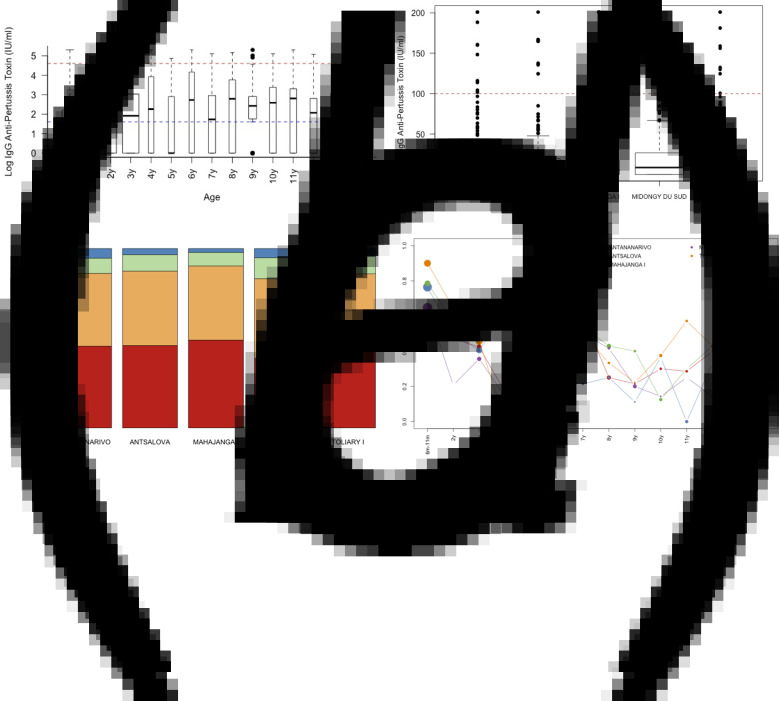
Age and geographic seropositivity results. Serum samples were tested for the presence of IgG anti-pertussis toxin by (a) age profile with a blue dashed line indicating <5 IU/ml and red dashed line for 100 IU/ml, (b) location, (c) distribution by seroprotection group per location, and (d) the proportion who are seronegative by age and location.

To address this, we analysed sera samples collected from 1078 individuals across five districts (see Fig. 1b, Materials and methods). Samples were made available as part of a polio surveillance project which focused on these districts. In total, 1078 sera samples were analysed for the presence of anti-PT IgG antibodies (see Table 2). In total, 45.36% of the population tested demonstrated anti-PT IgG titres of <5 IU/ml (see Fig. 2a). The highest percentage of negative serum by age group was the 6–11 months group at 68% (95% CI 61.67–73.69%; 155/228; *p* < 0.0001 *vs.* total). The proportion of the population presenting anti-PT IgG levels compatible with distant exposure to *B. pertussis* (anti-PT IgG level 5–39 IU/ml) was 41% (95% CI 38.10–43.96%; 442/1078) with the lowest percentage found in the 6–11 months group at 27.19% (95% CI 21.83–33.31%; 62/228; p< 0.0001 *vs.* total, Table 2). In terms of recent infection/vaccination, the proportion of the population presenting anti-PT IgG levels compatible with recent contact to *B. pertussis* (anti-PT IgG levels in the range of 40–99 IU/ml) was 9.46% (95% CI 7.85–11.35%; 102/1078) with the lowest percentage found in the 6–11 months group at 3.07% (95% CI 1.49–6.20%; 7/228; *p* = 0.0009 *vs.* total, Table 2) regardless of location. The proportion of the population presenting anti-PT IgG levels compatible with acute infection (anti-PT IgG levels 100 IU/ml) was low, at 4.17% (95% CI 3.13–5.53%; 45/1078) and lowest for 6–11 month group, but was not significantly different from other age groups.

**Table 2.** Distribution by age group and by anti-PT IgG titre categories of sera collected in 2016

Age groups	Number/total number % and (95% CI) anti-PT IgG (IU/ml)			
	<5	(5–40)	(40–10)	100
6–11m	155/22, 67.98% (61.67–73.69)	62/228, 27.19**%** (21.83–33.31)	7/228, 3.07% (1.49–6.20)	4/228, 1.75% (0.68–4.42)
3–4y	179/394, 45.43% (40.58–50.36)	161/394, 40.86% (36.11–45.78)	39/394, 9.89% (7.32–13.24)	15/394, 3.80% (2.32–6.18)
5–6y	30/64 46.87% (35.17–58.92)	21/64, 32.81% (22.57–45.00)	9/64, 14.06% (7.57–24.61)	4/64, 6.25% (2.45–14.99)
7–8y	30/76, 39.47% (29.24–50.71)	29/76, 38.15% (28.05–49.39)	12/76, 15.78% (9.26–25.60)	5/76, 6.57% (2.84–14.49)
9y	14/41, 34.14% (21.55–49.45)	16/41, 39.02% (25.65–54.27)	8/41, 19.51% (10.23–34.01)	3/41, 7.31% (2.51–19.42)
10y	14/61, 22.95% (14.19–34.91)	41/61, 67.21% (54.72–77.66)	3/61, 4.91% (1.34–13.49)	3/61, 4.91% (1.34–13.49)
11y	12/44, 27.27% (16.34–41.84)	23/44, 52.27% (37.93–66.24)	5/44, 11.36% (4.95–23.97)	4/44, 9.09% (3.59–21.15)
12–13y	29/90, 32.22% (23.46–42.43)	47/90, 52.22% (42.02–62.23)	9/90, 10.00% (5.35–17.92)	5/90, 5.55% (2.39–12.35)
14–15y	26/80, 32.5% (23.24–43.35)	42/80, 52.50% (41.69–63.07)	10/80, 12.50% (6.93–21.50)	2/80, 2.50% (0.44–8.66)
Total	489/1078, 45.36% (42.41–48.34)	442/1078, 41.00% (38.10–43.96)	102/1078, 9.46% (7.85–11.35)	45/1078, 4.17% (3.13–5.53)

m, months; y, years; IgG, immunoglobulin G; IU, international unit; CI, confidence interval.

### Geographic heterogeneity

By district, Midongy Atsimo had the lowest percentage of individuals with anti-PT IgG levels (<5 IU/ml) (see Fig. 2b and c) (MDG < TOL *p* = 0.0235; MDG < CUM *p* = 0.0408). The values in the four other districts were fairly similar across age classes. Table 3 shows an analysis of the distribution of anti-PT IgG by district in children aged 6–11 months. The results showed that a large proportion of children that did not mount an immune response against pertussis antigen (<5 IU/ml) were from Toliary I (90%; 95% CI 69.90–98.22%; 18/20; *p* < 0.0430 *vs.* total, Fig. 2c). Similarly, the lowest proportion of children with distant *B. pertussis* infection (5–40 IU/ml) was also in Toliary I (5%; 95% CI 0.26–23.61%; 5/20; *p* < 0.0350 *vs.* total). Compared with administrative estimates of vaccination coverage for these districts, Midongy Atsimo had the highest vaccination coverage based on administrative data for all three doses (see Supplementary Data File 1). However, given the difficulty in assessing actual coverage from reported data (doses divided by the target population), these results may be the result of errors in reporting [30]. Regardless, there was a large proportion of children who were not protected across all districts analysed (Fig. 2d).

**Table 3.** Distribution by districts, showing the result of anti-PT IgG titre categories, of sera collected among children 6–11month in 2016

Districts	Number/total number % and (95% CI) anti-PT IgG (IU ml/1)			
	<5	(5–40)	(40–100)	100
Antsalova	39/51 76.47% (63.24–86.0)	11/51, 21.57% (12.49–34.63)	0	1/51, 1.96% (0.10–10.30)
CUA	32/58 55.17% (42.45–67.25)	21/58, 36.21% (25.05–49.07)	4/58, 6.90% (2.71–16.43)	1/58, 1.72% (0.09–9.14)
CUM	11/14, 78.57% (52.41–92.43)	2/14, 14.29% (2.54–39.94)	0	1/14, 7.14% (0.37–31.47)
Midongy Atsimo	55/85, 64.71% (54.11–74.03)	27/85, 31.76% (22.84–42.27)	3/85, 3.53% (0.96–9.87)	0
Toliary I I	18/20, 90% (69.90–98.22)	1/20, 5% (0.26–23.61)	0	1/20, 5% (0.26–23.61)
Total	155/228, 67.98% (61.67–73.70)	62/228, 27.19% (21.83–33.31)	7/228, 3.07% (1.49–6.20)	4/228, 1.75% (0.68–4.42)

CUA, Urban Commune of Antananarivo; CUM, Urban Commune of Mahajanga; IgG, immunoglobulin G; IU, international unit; CI, confidence interval.

### Mathematical model of seropositivity in each of the five districts

**Fig. 3.**
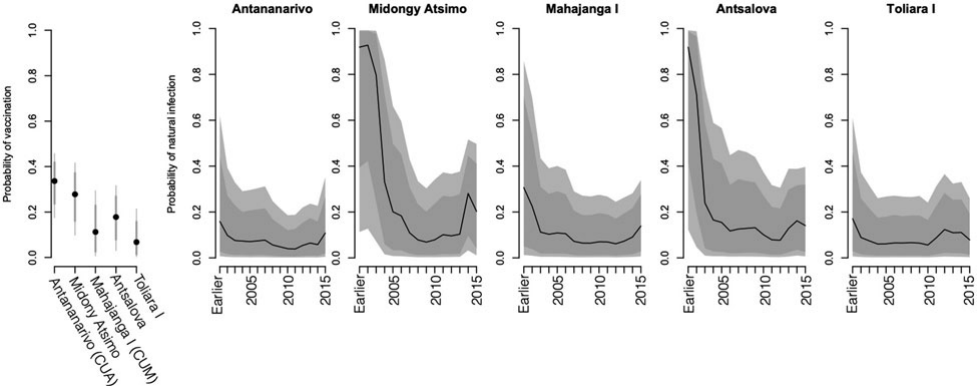
Determinants of seropositivity estimates of the probability of becoming seropositive by vaccination (*y*-axis, first panel) in each of the five communes; and associated force of infection (*y*-axis, panels 2–6) across years (*x*-axis) for each of the five locations (panel titles); showing 95% (light grey) and 80% (dark grey) credible intervals from the posterior distributions of associated rates. See text for model assumptions; estimates are robust to altering the prior distributions. The pattern of decay in the force of infection broadly matches the reported pattern from national scale reporting (Fig. 1a).

The model described above indicates spatial heterogeneity in the opportunity of becoming seropositive by vaccination (Fig. 3, left panel), with the highest coverage estimated to occur in Antananarivo, the capital; and lowest in Toliara I; but there is considerable uncertainty in these estimates (vertical lines indicating credible intervals). There is also substantial spatial and temporal variability in the force of infection, or probability that susceptible individuals were naturally infected (Fig. 3, panels 2–6). The temporal trajectory in the force of infection broadly recapitulates patterns reported in national case data (Fig. 1a), as expected, since the force of infection should reflect the product of the number of infected individuals, the probability that they encounter susceptible individuals, and the magnitude of transmission. If vaccination coverage has increased through time, this may result in upward biases in estimates of the force of infection at later time points suggested particularly in Midongy Atsimo; if immunity wanes, this will result in underestimates of the force of infection across time points experienced by individuals old enough to have experienced waning.

### Individual-level analysis

**Fig. 4.**
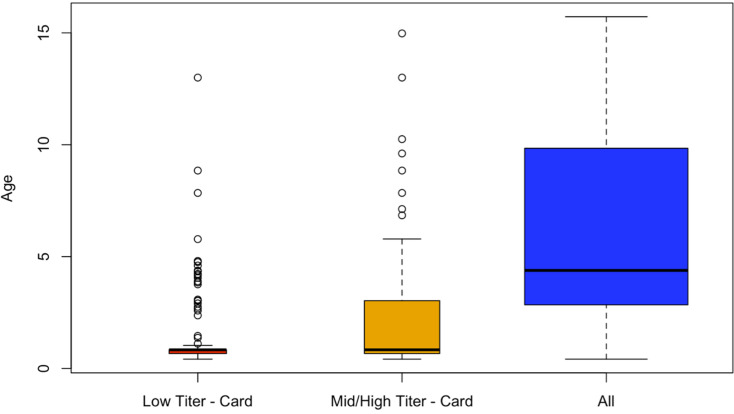
Individual-level analysis. For a small number of individuals who possess a vaccination card, the majority were not serologically protected (titre values below 5). We analysed the age profile of these individuals relative to others who also possessed a vaccination card and the study population. Those who had a low titre level and a vaccination card were on average 1.47 years whereas those who had a low titre level but did not possess a vaccination card were on average 6 years old (*P* < 0.001).

A small number of individuals (*n* = 229) had evidence of being vaccinated by owning a vaccination card where routine vaccinations are recorded. For the remaining individuals, vaccination history could not be confirmed with these data. The majority of these individuals (58%) were seronegative (anti-PT IgG titres of <5 IU/ml) despite likely being vaccinated. Only 21% (47/229) of children with the history of vaccination have at least the expected titre level from completing the entire three-dose regime (³20 IU/ml). Among the remaining 79% (182/229) vaccinated but anti-PT IgG titre <20 IU/ml, astonishingly, 74% (134/182) were children in the age group 6–11 months. Few individuals (3%) had titres indicative of recent infection or vaccination (100 IU/ml) [21], with only 2% (4/162) in children aged 6–11 months. Among likely vaccinated but seronegative individuals, the majority, 80% (107/133), were between 6 months and 1 year old which is a particularly high-risk population (Fig. 4).

## Discussion

In this study, we assessed the immune status of children in Madagascar against pertussis in five districts across the country. The majority of children, particularly those in the highest risk group (aged 6–11 months) were not protected (IgG titre levels below 5 IU/ml) against pertussis 67.98% (95% CI 61.67–73.69%; 155/228; *p* < 0.0001 *vs.* total). Although vaccination history was only provided for a small number of individuals, we also found that a large proportion of likely vaccinated individuals (as indicated by the possession of a vaccine card) (58.08%; 133/229) were also seronegative. In either case, results suggest that children are not being vaccinated, completing their entire dosing schedule, or being immunised by vaccination. The latter might occur for a number of reasons, e.g. if there were issues with the vaccine cold chain, or if children are immunosuppressed and not mounting an adequate immune response to vaccination. Spatial differences in seronegativity did not correlate with district-level estimates of vaccination coverage based on administrative data (Pearson's correlation coefficient: −0.95, *p* = 0.012). This lack of relationship might emerge for the reasons described above (cold-chains issues, immunosuppression), or because of inaccuracies in the vaccination coverage data [21, 30]. In addition, malnutrition is prevalent in Madagascar and it is known that levels of lower levels of antibodies are produced after vaccination in severely malnourished children [21, 30–32].

We further found evidence of natural infection, both from a catalytic model and directly in the serological data, in all age groups across the five districts. Although the number of districts limited the generalizability of our analysis, they nonetheless provide an assessment of seropositivity and evidence of natural infection. There was no clear age or spatial clustering of infection suggesting that *B. pertussis* is circulating widely across Madagascar, despite the fact that the reported cases to the WHO (Fig. 1a) suggest limited transmission. However, our study was limited to a small number of districts in Madagascar (5 out of 115) limiting our ability to conduct a more thorough analysis of pertussis across the country. Further, it is not clear how representative the five districts analysed are of the national patterns of transmission. The samples analysed were not systematically collected and thus may not be representative of serological patterns in other areas of the country. Obtaining additional serum samples, clinical and biological data from across the country would enable such an analysis.

We used a catalytic model to estimate the force of infection and this model made a number of important assumptions. In particular, the dominant source of pertussis immunity in children aged <9 months was assumed to be vaccination rather than infection, which could result in underestimation of circulation of the infection. Strikingly, even with this potential bias in the direction of over-estimation, estimates of immunization by vaccination are remarkably low, highlighting the importance of strengthening vaccination programmes for pertussis in Madagascar. All five districts show an increase in the force of infection towards the end of the time-series (Fig. 3). Given the design of the model and its inability to distinguish changes in natural *vs.* vaccine-induced immunity, this might reflect an increase in the circulation of pertussis in these later years, but it might also reflect an increase in vaccination coverage occurring later on in the time-series since the model framework does not allow vaccination coverage to change through time. Further data on both health systems function and serostatus could help explore these different explanations.

This study provides some evidence indicating that whooping cough has been circulating throughout at least five districts in Madagascar. Lack of data on vaccination coverage in some areas of Madagascar and the lack of vaccine booster in children over 3 years old are likely to be important components of this immunity gap; but the quality of vaccine delivery as well as immunogenicity of the vaccine in communities across the island are open questions, and would be an important direction for further investigation. This study also demonstrates the importance of seroepidemiological surveillance of whooping cough and encourages its implementation in Madagascar.

## Conclusions

Although pertussis is a highly contagious infectious disease that is an important cause of mortality and morbidity worldwide, ineffective case surveillance systems and vaccination coverage estimates make understanding the circulation of natural infection and effectiveness of vaccination difficult. Particularly in low-income settings, such as Madagascar, there is a limited understanding of the epidemiology of pertussis. Here, we analysed serological data from five districts throughout Madagascar and identified the patterns of seropositivity across ages and locations. Overall, we found that in the districts analysed, many children, particularly those in the high-risk 6–11 months age group, were not seropositive. Immunization programmes within the country should be strengthened to improve the proportion who are seropositive.

## Data Availability

Data and code are available at: https://github.com/apwez/Pertussis_Madagascar

## Annex

Performance of commercial enzyme-linked immunosorbent assays for detection of antibodies to *Bordetella pertussis*

**TABLE 4.** Sensitivity and specificity of commercial IgG ELISAs compared to an anti-PT IgG in-house ELISA^*a*^

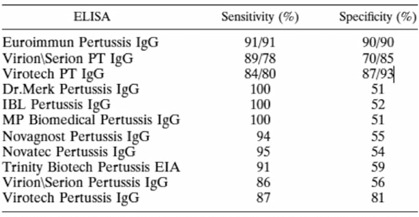

a Indeterminate results according to the kit's insert were interpreted as positive. Sensitivity and specificity of kits measuring anti-PT were also estimated when the in-house ELISA cutoff (≥40 IU/ml) was used (second number where two numbers are given).
